# Ribosome heterogeneity and specialization in musculoskeletal physiology and pathology

**DOI:** 10.1093/jbmrpl/ziag018

**Published:** 2026-02-02

**Authors:** Alzbeta Chabronova, Guus G H van den Akker, Tim J M Welting, Mandy J Peffers

**Affiliations:** Department of Musculoskeletal Ageing Science, University of Liverpool, Liverpool, L78 TX, United Kingdom; Laboratory for Experimental Orthopaedics, Department of Orthopaedic Surgery, Maastricht University, Maastricht, 6229 HX, The Netherlands; Laboratory for Experimental Orthopaedics, Department of Orthopaedic Surgery, Maastricht University, Maastricht, 6229 HX, The Netherlands; Department of Musculoskeletal Ageing Science, University of Liverpool, Liverpool, L78 TX, United Kingdom

**Keywords:** protein synthesis, translation regulation, ribosome heterogeneity, ribosome specialization, ribosomal RNA, 2′-*O*-methylation, pseudouridylation, snoRNAs, ribosomal proteins, ribosome-associated proteins

## Abstract

Musculoskeletal (MSK) tissues are highly dynamic systems that rely on tightly regulated protein synthesis to maintain homeostasis and structural integrity, adapt to physiological stimuli, and respond to injury. The deregulation of protein synthesis is implicated in a wide range of MSK pathologies. At the core of protein synthesis are ribosomes, complex molecular nanomachines that translate mRNAs and generate proteins. Once considered uniform entities passively exerting their function, ribosomes are now recognized to be heterogeneous in their composition and capable of specialized functions. These emerging concepts of ribosome heterogeneity and specialization are increasingly recognized as key regulators of physiological and pathological cellular processes across fields. Although the MSK field has yet to fully embrace and integrate ribosome-centered research, accumulating evidence suggests that ribosome heterogeneity and specialization might have profound implications for MSK (patho)biology. In this review, we summarize the emerging data across MSK tissues (bone, skeletal muscle, articular cartilage, tendons, and ligaments), highlighting the roles of ribosomes in supporting development, maintaining homeostasis, and facilitating cellular and tissue functions and adaptations, but also driving pathological changes and disease progression. Furthermore, we also outline recent key technological and methodological advances that are critical for uncovering the full scope, significance, and dynamic regulation of ribosome heterogeneity and specialization in MSK (patho)biology. As the field moves forward, ribosome-centered research holds great promise in revealing new mechanisms underlying MSK biology and identifying novel therapeutic targets.

## Graphical Abstract

**Graphical Abstract:**
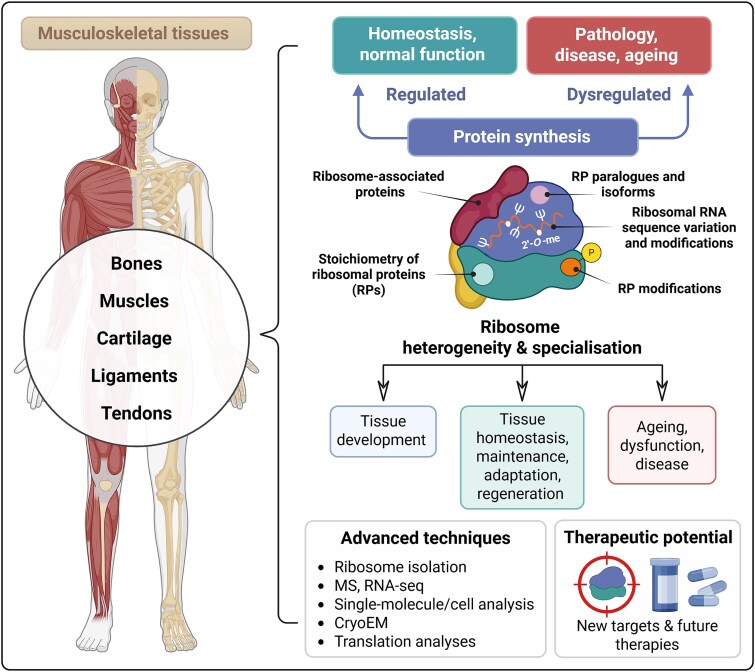

## Introduction

The musculoskeletal system (MSK) is the biggest human organ system, integrating bones, skeletal muscles, articular cartilage, ligaments, and tendons. It provides the structural support, enables locomotion and force generation, protects internal organs, regulates mineral homeostasis, and facilitates hematopoiesis and metabolic functions. It is a core component of functional capacity, strength, and endurance.

Protein synthesis and its precise dynamic regulation are critical for MSK homeostasis and physiological functions. They facilitate skeletal muscle maintenance and hypertrophy,^1^ bone matrix formation and remodeling,^2^ as well as extracellular matrix (ECM) production and turnover in cartilage, tendons, and ligaments.^3,4^ The dysregulation of protein synthesis is associated with a plethora of pathologies, including muscle atrophy,^5^ sarcopenia,^6^ Duchenne muscular dystrophy (DMD),^7^ osteogenesis imperfecta,^8^ osteopenia and osteoporosis,^9^ osteoarthritis (OA),^4^ and others.

Within the cell, proteins are synthesized in a multi-step gene expression process, during which the information encoded in genes is transcribed into mRNAs and then translated by ribosomes into functional proteins. As mRNAs are easier to quantify, the analyses of transcriptomes have dominated the field for years. However, accumulating evidence demonstrates a discordance between mRNA levels and protein abundances. The mRNA level is not a definite proxy for protein abundance; in fact, the relationship between mRNA and protein is complex, non-linear, and varies significantly from gene to gene. With that, the significance of gene expression regulation at the level of translation has been increasingly recognized over recent years.^10^ Several layers of translation regulation center around the ribosome. The ribosome is a complex cellular nanomachine that decodes the genetic code carried by mRNAs into chains of amino acids, thus forming polypeptides and eventually proteins. While the rate of ribosome biogenesis (thus the total number of ribosomes) determines the global translational capacity of the cell, more nuanced, and arguably intriguing mechanisms can lead to translation regulation at the level of individual mRNAs. Recent data show that differences in ribosome composition, also known as ribosome heterogeneity, can affect ribosomes’ interactions with specific mRNAs, regulate translation initiation, elongation speed, and translation fidelity, thus allowing for rapid adjustments in the levels of specific proteins in response to various cellular stimuli.^11–16^

Ribosome-centered translation regulation is an emerging and rapidly growing area of research.^17,18^ In 2009, a Nobel Prize in Chemistry was awarded to V. Ramakrishnan, T.A. Steitz, and A.E. Yonath for their groundbreaking high-resolution atomic maps of ribosomal subunits explaining fundamental mechanisms of protein synthesis.^19^ Since then, a remarkable series of ribosome structures has been solved (^20–22^ and extensively reviewed^20–26^), uncovering the ribosome’s function in translation and providing a framework for research on ribosome heterogeneity and functional specialization. Combining these structural insights with data generated by various recently developed methods, we are uncovering novel mechanisms of ribosome-mediated translational regulation with important consequences for human physiology and disease.^14–16,27^ Nevertheless, ribosome research in the MSK field remains relatively limited. In this review, we summarize the current data on ribosomes in MSK biology and highlight potential directions for future investigations, hopefully inspiring future research in this up-and-coming research area.

## A eukaryotic ribosome and its biogenesis

**Figure 1:**
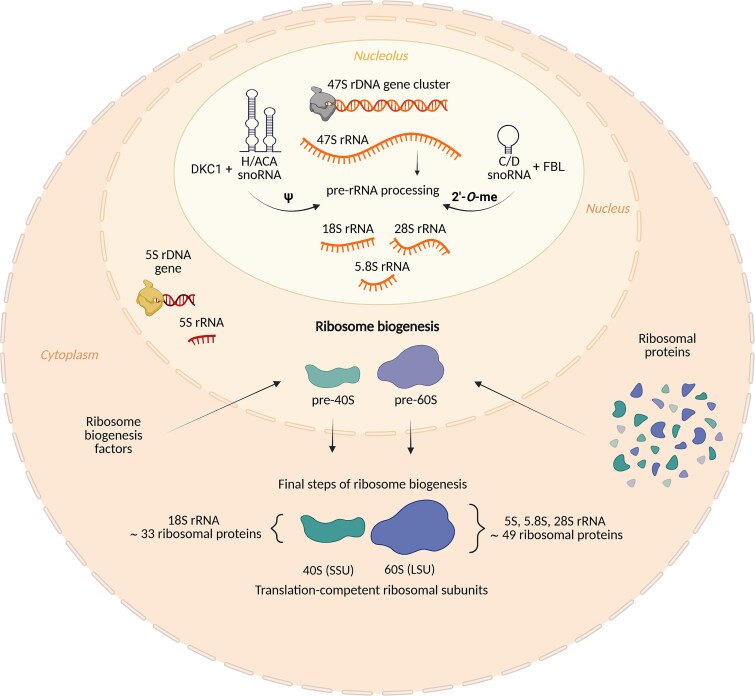
The essentials of eukaryotic ribosome biogenesis. Ribosome biogenesis is a multi-step process that spans three cellular compartments—nucleolus, nucleoplasm, and cytoplasm. The main steps are: (i) rDNA transcription generating 47S pre-rRNA and 5S rRNAs; (ii) 47S pre-rRNA processing, 18S, 5.8S, and 28S maturation; (iii) post-transcriptional modifications (PTMs) of rRNAs by snoRNAs; (iv) import of ribosomal proteins (RPs) and ribosome biogenesis factors into the nucleus, assembly of pre-ribosomal subunits, quality check, nucleolar export; (v) ribosome maturation and quality control. SnoRNAs are small nucleolar RNAs, whose main function is to guide site-directionality of the rRNA PTMs and, in some cases, also aid specific 47S pre-rRNA processing steps—eg, U3 snoRNA or RMRP (the ribonuclease mitochondrial RNA processing). Abbreviations: 2′-*O*-me, ribose 2′-*O*-methylation; C/D snoRNA, box C/D small nucleolar RNA; DKC1, pseudouridine synthase dyskerin; FBL, methyltransferase fibrillarin; H/ACA snoRNA, box H/ACA small nucleolar RNA; LSU, large ribosomal subunit; RP, ribosomal protein; rRNA, ribosomal RNA; SSU, small ribosomal subunit; ψ, pseudouridylation. Figure created using BioRender.

Human ribosome consists of four ribosomal RNAs (rRNAs; 18S, 5.8S, 28S, and 5S) and up to 80 ribosomal proteins (RPs) that are assembled into a small ribosomal subunit (40S) and a large ribosomal subunit (60S).^21^ The process of ribosome biogenesis is one of the most energy-consuming cellular processes, and several hundred ribosome biogenesis factors^28–30^ and small nucleolar RNAs (snoRNAs)^31^ carefully regulate all steps of this intricate process^32^ (summarized in Figure 1). Furthermore, many regulatory mechanisms are in place to control and fine-tune individual steps of ribosome biogenesis, regulating not only the total number, but also the composition (and thus the functional specialization) of ribosomes in the cell at any given time to reflect immediate cellular needs.

## Ribosome heterogeneity

Right after the discovery of ribosomes in the mid-1950s,^33^ scientists considered the possibility of ribosome heterogeneity in terms of their composition, activity, and function. However, the experimental methods necessary to test these hypotheses were lacking at the time, and the idea was largely abandoned. Consequently, the prevailing dogma describing ribosomes as homogeneous complexes that passively translate mRNAs into proteins dominated the scientific community.

By the early 2000s, however, advances in crystallization and cryogenic electron microscopy revolutionized the field by solving the ribosome structure at the atomic level and paved the way for more focused and detailed ribosome research.^19^ Together with advances in next-generation sequencing, quantitative mass spectrometry, and data analysis pipelines, this enabled researchers to revisit the hypothesis of ribosome heterogeneity. Since then, undeniable evidence for ribosomal compositional diversity—ribosome heterogeneity—has emerged, along with data supporting the functional specialization of heterogeneous ribosomes—ribosome specialization.^11,12,34^

**Figure 2:**
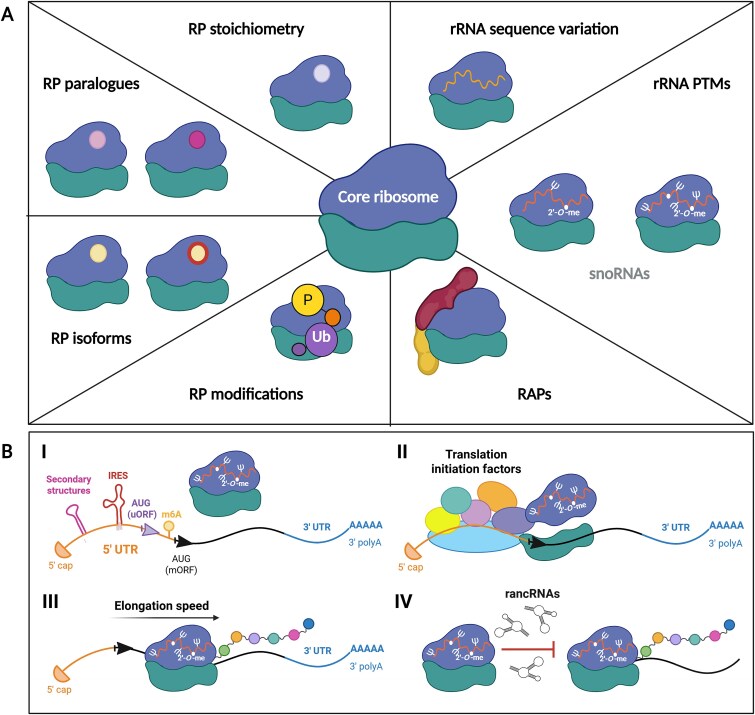
(A) Sources of ribosome heterogeneity. The ribosome is in the middle (40S in green, 60S in blue). Each section represents a different level of ribosome heterogeneity. Alternative coloring indicates variation (rRNA sequence variants, RP paralogues, isoforms). P represents phosphorylation, Ub symbolizes ubiquitination. (B) Proposed mechanisms of ribosome specialization. (I) Ribosomes interact with mRNAs 5′ untranslated regions (5′ UTRs) which contain specific secondary structures, IRES, upstream ORFs (uORFs), or modified nucleotides (eg, N6-methyladenosine) that regulate translation of given mRNA.^58^ (II) Ribosomes’ interactions with specific RAPs also affect translation. For example, several paralogues and isoforms of translation initiation factors exist in eukaryotes, and data suggest they have different affinities for subsets of mRNAs, thus affecting their translation.^59^ (III) Regulation of translational elongation might also play a role in translation regulation.^60^ (IV) Interactions between ribosomes and a group of ribosome-associated non-coding RNAs (rancRNAs) have been described and suggested to repress translation.^61^ Abbreviations: 2′-*O*-me, ribose 2′-*O*-methylation; RAP, ribosome-associated protein; RP, ribosomal protein; rRNA, ribosomal RNA; snoRNA, small nucleolar RNA; ψ, pseudouridylation. Figure created using BioRender.

Ribosome heterogeneity can arise from alterations in RP stoichiometry, the incorporation of RP paralogues or isoforms, post-translational modifications of RPs, interactions with various ribosome-associated proteins (RAPs),^11,12,35,36^ rRNA sequence variation,^37,38^ and rRNA post-transcriptional modifications (PTMs)^39–41^ (Figure 2A). While the total number of ribosomes directly impacts cellular translation capacity, ribosome heterogeneity can influence the cellular proteome and phenotype in a more specific and targeted way.^11,12^ One of the suggested mechanisms by which heterogeneous ribosomes can exert specialized functions is through the control of the translation efficiency of specific mRNAs (Figure 2B). Ribosome heterogeneity has been documented at the level of different species,^35,39,42–44^ developmental stages,^42,45–50^ tissues,^12,27,39,41,51^ within a single cell,^34,50^ in disease,^12,39,52–55^ and distinct growth conditions.^50,56,57^ The examples of ribosome specialization are more limited; nevertheless, the evidence is emerging. In the following sections, we summarize the key evidence and novel findings supporting the concepts of ribosomal heterogeneity and specialization.

### Ribosomal proteins

RP-based ribosome heterogeneity arises from alterations in RP stoichiometry, the incorporation of RP paralogues or isoforms, RP post-translational modifications, and interactions with various RAPs.^11,12,35,36^

### RP stoichiometry

RP stoichiometry refers to the precise number and ratio of different core RPs incorporated within the ribosome. Differential expression of RPs across different tissues, cell types, or various (patho)physiological conditions was reported^12,50^ suggesting the regulation of RPs stoichiometry within ribosomes. However, a change in RP expression (transcript and protein levels) does not always lead to the differential stoichiometry.^44,49,51^ Therefore, proteomic analyses of purified ribosomes are necessary to uncover the extent and consequences of RP-mediated ribosome heterogeneity.

Tissue-specific differences in RP stoichiometry were demonstrated recently by analyzing ribosomes purified from 14 adult mouse tissues (lungs, kidneys, adrenal glands, liver, small intestine, spleen, testis, heart muscle, quadriceps skeletal muscle, cortex, hippocampus, olfactory bulbs, cerebellum, and retina) using MS-based quantitative proteomics.^51^ In fact, RPs clustered into four groups: stable, variable across specific tissues, variable across all tissues, and tissue-specific.^51^ Marked differences in RP abundance were also measured in 80S monosomes from nine different tissues in mice (brain, lung, spleen, kidney, fat, testis, heart, liver, and skeletal muscle),^62^ further corroborating the tissue-specificity of RP-mediated ribosome heterogeneity.

Slavkov and colleagues quantified RPs in the ribosomes of the budding yeast *Saccharomyces cerevisiae* and mouse embryonic stem cells (mESCs). They demonstrated that RPs’ stoichiometry in wild-type *S. cerevisiae* and mESCs differs between monosomes and polysomes, and that it changes depending on specific growth conditions. Deletions of RPs enriched in polysomes decreased cellular fitness significantly more than the deletion of RPs enriched in monosomes.^50^ Heterogeneous stoichiometry of RPs in translating ribosomes of mESCs was corroborated in another study, which also showed that heterogeneous ribosomes preferentially translate a subset of mRNAs. This level of regulation was, at least partially, mediated by translation initiation from internal ribosome entry sites (IRES) elements.^63^ Ribosomal protein abundances were also measured in human embryonic stem cells (hESCs) differentiated down the endoderm and mesoderm lineages. Compared to undifferentiated hESCs, changes in polysomal RP composition were detected over the course of cellular differentiation.^49^ Solvent accessibility experiments indicated that heterogeneous RPs are located on the surface of the ribosome, some in the proximity of the mRNA entry and exit channels. Among the RPs that exhibited an altered abundance within polysomes during cellular differentiation were proteins previously identified as substoichiometric in actively translating ribosomes in mESCs,^63^ supporting the observation of their dynamically regulated stoichiometry. RPL10A (uL1^64^) was one of the regulated RPs. Its stoichiometry was upregulated in the polysomes of the primitive streak cell types and then progressively declined during mesoderm differentiation toward the sclerotome. A *Rpl10a* loss-of-function mouse exhibited striking early mesodermal phenotypes, and polysomal profiling revealed decreased translation of mesoderm regulators. RPL10A was also shown to regulate canonical and non-canonical Wnt signaling during stem cell differentiation and in the developing embryo.^49^ Altogether, these results show that RPs’ stoichiometry and ribosome heterogeneity control differentiation and development through the specialized translation of key signaling networks.

Furthermore, a recent study analyzed the kinetics of RPs’ turnover in assembled ribosomes isolated from mouse liver in vivo.^65^ Interestingly, turnover rates were significantly different between RPs. The majority of RPs appeared to be integrated into the ribosome during initial assembly and was degraded together as a unit by ribophagy. However, a subset of RPs, located predominantly at the ribosome periphery, was replaced multiple times during the lifespan of the ribosome, presumably by an exchange with a free RP cytoplasmic pool. Dietary signals impacted the rates of both new ribosome assembly as well as the exchange of individual components.^65^ Altogether, these results suggest that RP composition, previously considered to be quite invariable once the ribosome is assembled, may be capable of relatively rapid remodeling in response to changing cellular needs.

### Isoforms

Ribosomal protein isoforms, slightly different protein products generated from the same gene (*eg,* through alternative splicing), are another source of ribosome heterogeneity. While the differential expression and abundance of RP isoforms were reported, especially in the context of cancer,^66–70^ data on the differential incorporation of these isoforms into ribosomes is mostly missing. A recent paper studying glioblastoma (GBM) identified a novel isoform of RPL22L1 (eL22L1), which was termed RPL22L1b. The levels of RPL22L1b were significantly higher in the core of the GBM tumor when compared to its periphery. Importantly, this was recapitulated in ribosomes purified from GBM sphere lines with core and edge phenotypes. The authors demonstrated that an acidic microenvironment—found in the tumor core—promotes the expression of SRSF4, which then shifts RPL22L1 splicing toward the RPL22L1b isoform in human gliomas in vitro and in situ. Manipulation of RPL22L1 isoforms in patient-derived GBM neurospheres affected proliferation in an acidified medium, where the expression of the RPL22L1b isoform offered a proliferation and survival advantage.^69^ This study provided insights into the regulation of RP isoform production and functions. Nevertheless, the research on ribosome heterogeneity and specialization mediated by the incorporation of RP isoforms is still in its infancy, and further research is needed.

### Paralogues

In higher eukaryotes, most RPs are present as single-copy genes; however, a handful of RPs have paralogues that emerged through duplication events. The differential expression of paralogues was reported in numerous studies (reviewed recently in^15^). Nevertheless, in most cases, their biological significance in the context of ribosome heterogeneity and specialization remains to be investigated.

Even though paralogues are almost identical in sequence, their functional characteristics can be different. Data from yeast show that paralogues of RPL7 (uL30)—RPL7A and RPL7B, which differ in the acetylation of their N-terminal domains, differentially regulate ribosome biogenesis and translation characteristics.^71^ Under normal conditions, yeast generated ribosomes with the predominant, hypoacetylated paralogue, RPL7A. The RPL7A-ribosomes then preferentially translated genes with short open reading frames (ORFs). On the other hand, exposure to drugs increased the production of ribosomes with the hyper-acetylated paralogue RPL7B, which led to the increased translation of long ORFs, many of which encode cell wall proteins that are linked to drug resistance.^71^

In higher eukaryotes, an RP paralogue switching was reported in the gonads of *Drosophila melanogaster*,^44^ and a testis-specific paralogue, RPL39L (eL39L) was shown to play a critical role in spermatogenesis in mice.^62^ Cryogenic electron microscopy (cryo-EM) structures of ribosomes were generated and provided mechanistic insights into how RP paralogues might lead to ribosome specialization by affecting ribosome interactions with mRNAs,^44^ or by altering the conformation of the nascent polypeptide exit tunnel.^62,72^ Another study demonstrated that RPS27 (eS27) and RPS27l (eS27L)—containing ribosomes associate preferentially with different transcripts,^73^ further supporting the notion of functional ribosome specialization mediated by RP paralogues.

### Post-translational modifications of RPs

Protein post-translational modifications are covalent modifications that can alter the stability, subcellular localization, and interaction partners of the modified protein. Ribosomal proteins harbor a variety of modifications, including phosphorylation, ubiquitination, methylation, acetylation, or hydroxylation.^74^ Again, several studies have reported differential RP modifications in different (patho)physiological conditions (reviewed in^74^), however, only a few have investigated them in the context of ribosome heterogeneity and specialization. Two studies showed that the site-specific ubiquitylation of RPs, including RPS2 (uS5), RPS3 (uS3), RPS10 (eS10), and RPS20 (uS10), is important for ribosome stalling at poly(A) sequences and translation fidelity, thus aiding protein quality control to minimize the production of aberrant proteins.^75,76^ A ribosome phosphoproteomic analysis identified 46 phosphosites in the RPs of mammalian cells. The phosphorylation of serine 38 (pS38) in RPL12 (uL11) was selected for functional validation, as it is conserved across species, and it was shown that its phosphorylation status affects the translation of mitosis-related mRNAs.^77^ Another study reported that the hydroxylation of RPL27a (uL15) at its His39 residue, located near the CCA terminus of the E-site-bound tRNA, is regulated by hypoxia. A RPL27a mutant, incapable of hydroxylation, exhibited reduced translational activity and the altered translation of specific mRNAs.^78^

### Ribosome-associated proteins

Ribosome-associated proteins are proteins transiently or permanently associated with the ribosome that regulate various aspects of ribosome activity and translation. In one of the first studies of its kind, Simsek and colleagues set up a ribosome affinity purification method and identified hundreds of proteins (~400) associated with ribosomes in mESCs. These included the expected components of the canonical translation machinery, including translation initiation, elongation, and termination factors, as well as RNA helicases and factors involved in protein folding. In addition, proteins controlling metabolism and the cell cycle, as well as proteins involved in mRNA processing, splicing, transport, and many others, were also found.^79^ Most of the identified RAPs were directly bound to the ribosome, and only a small fraction was brought to the ribosome by interactions with mRNAs or nascent peptide chains.^79^ These findings revealed a broad landscape of RAPs that contribute to ribosome heterogeneity and may also drive the functional diversification of ribosomes. To this end, the authors also demonstrated that the metabolic enzyme pyruvate kinase muscle interacts with subpools of endoplasmic reticulum (ER)-associated ribosomes and facilitates the translation of ER-related mRNAs.^79^

Recently, a label-free methodology called RAP identification by affinity to sulfhydryl-charged resin (RAPIDASH) was developed to enrich ribosomes and associated proteins. Using this method, 566 RAPs were identified across the limbs, forebrain, and liver tissues in developing mouse embryos.^27^ Among these, Dhx30 and Llph were identified as two forebrain-specific RAPs important for neurodevelopment, with Llph possibly regulating the elongation and translation of long mRNAs.^27^ In addition, the authors also reported a dynamic ribosome RAPs remodeling during immune cell activation and macrophage stimulation.^27^

In another study, 145 polysome-associated proteins were identified in HEK293 and HeLa cells, 84 of which have not been described previously.^77^ The cytosolic polysomes of human cardiomyocytes (CMs) were shown to associate with mitochondrial ribosomal proteins (MRPs).^80^ The most notable was the increased abundance of MRPS15 in the polysomes of CMs exposed to ER stress. MRPS15 was identified as an activator of IRES-mediated translation, and MRPS15-containing ribosomes were shown to preferentially associate with mRNAs containing IRES elements. This was in line with the observed increase in IRES-mediated translation in ER-stressed CMs.^80^ Altogether, these studies highlight the diversity of the extended ribosomal proteome and its consequences for the fine-tuning of ribosome functions.

### Ribosomal RNAs

The rRNAs carry out the polypeptide-generating catalytic properties of the ribosome, serve as a binding sites for RPs, and facilitate interactions with RAPs;^81^ hence, they have a great potential to influence ribosome function. The rRNA-based ribosome heterogeneity arises from rRNA sequence variation and rRNA PTMs.

### rRNA sequence variation

The rRNA genes (rDNA) are present in hundreds of copies in tandem repeats spread across multiple chromosomal loci.^38,82^ Park and colleagues analyzed the sequencing data from the 1000 Genomes Project containing data from 2546 individuals from 26 populations, and reported great variability in rDNA copy number between individuals (ranging from 61 to 1590 copies per person). They also identified pervasive inter- and intra-individual nucleotide variation in rRNA genes. Similar data were obtained when analyzing 32 strains of the Mouse Genomes Project. Mouse and human shared several rRNA variant alleles, many of which were located at positions important for ribosome function. Analysis in the mouse demonstrated the differential expression of individual rRNA variants in different organs (brain, lung, liver, and ovary). Importantly, the rRNA variants were present in actively translating ribosomes, as shown by analyzing polysomes isolated from mouse mammary epithelial cells.^38^ Data on rRNA sequence variation, as well as tissue-specific expression, were corroborated by Rothschild and colleagues, who analyzed 30 individuals of diverse ancestral origins added to the 1000 Genomes Project in 2022.^83^ Their data also suggest that 28S variants generate ribosomes with distinct structures—further supporting the notion that rRNA sequence variation could lead to the production of functionally distinct ribosomes. Lastly, by analyzing 10 030 samples with clinical phenotypes from The Cancer Genome Atlas, they identified rRNA sequence variants associated with cancer.^83^

Distinct rRNA alleles are expressed by the malaria parasite *Plasmodium falciparum* in asexual and sporozoite stages^84,85^ or by zebrafish during their development.^37^ Another study showed that while all seven rDNA operons in the common laboratory *Escherichia coli* strain MG1655 were expressed at approximately equal levels in a rich growth medium, under stress conditions represented by nutrient limitation, the expression levels of individual rDNA operons were reduced to different extents.^86^ In *Vibrio vulnificus*, ribosomes containing specific rRNA variants facilitated the preferential translation of a subset of mRNAs important for a rapid adaptation to temperature and nutrient shifts.^87^

Although rDNA has traditionally been considered a “dark area” of the genome due to poor sequence coverage and annotation, advances in sequencing technologies and data analysis are now enabling more detailed studies. Consequently, future research is likely to reveal important insights into how rRNA sequence variation contributes to ribosome heterogeneity and specialization.

### PTMs of rRNAs

A complete map of chemical modifications of rRNAs in the human 80S ribosome was published in 2018. Using quantitative mass spectrometry, Taoka and colleagues identified 14 distinct types of rRNA modifications and detected up to 228 modified rRNA nucleotides.^88^ The rRNA PTMs can be categorized into three groups: (1) ribose methylations (2′-*O*-me), (2) pseudouridylation (Ψ), and (3) nucleobase modifications such as methylation (mN), acetylation (acN), and aminocarboxypropylation (acpN).^88^ 2′-*O*-me and Ψ are by far the most abundant ones, accounting for more than 100 modified nucleotides each.^41,88,89^ They are installed by snoRNP complexes. Within these complexes, snoRNAs guide the site-specificity/directionality of the modifications by base-pairing with the target rRNA sequence, while methyltransferase fibrillarin (FBL) or pseudouridine synthase Dyskerin (DKC1) catalyze the 2′-*O*-me and Ψ of the given rRNA nucleotide.^31,90^ High-throughput methods to systematically map 2′-*O*-me (RiboMethSeq)^91,92^ and Ψ (HydraPsiSeq)^89^ were published in 2015 and 2020, respectively, and significantly propelled the research in this field. On the other hand, the base modifications, installed by stand-alone enzymes, are much less abundant and more difficult to measure.^93,94^

The majority of rRNA PTMs are located in the interior of the ribosome, near the functionally important regions, including the decoding center, tRNA binding sites, peptidyl transferase center, intersubunit bridge, and polypeptide exit tunnel.^88^ Generally, these rRNA PTMs are highly evolutionarily conserved and invariably modified across species, cell types, and conditions,^41,88,93,95^ suggesting that they are indispensable for ribosome function. The substoichiometric, variable rRNA PTMs are more interesting in terms of ribosome heterogeneity. They are less conserved, frequently located on the periphery of the ribosome, and exhibit significant variability in their modification levels in different physiological and pathological conditions^41,88,93,95^ and are therefore believed to fine-tune ribosomal catalytic activity.

While the dynamic regulation of rRNA PTMs in various growth conditions, physiological, and pathological conditions or disease contexts is well documented, evidence directly linking rRNA PTM-mediated ribosome heterogeneity to ribosome functional specialization is more limited. A recent study reported site-specific changes in rRNA 2′-*O*-me levels in response to upstream MYC oncogene expression. The site 18S-Cm174 was identified as one of the most robustly MYC-regulated 2′-*O*-me sites. By knocking out (KO) *SNORD45C*, which guides the 2′-*O*-me of this particular nucleotide, the authors demonstrated that ribosomes depleted of 18S-Cm174 exhibit an altered translation of a subset of mRNAs linked to a cancer phenotype.^95^ The rRNA 2′-*O*-me were also shown to regulate ribosome translation programs in acute myeloid leukemia. In particular, 18S-Gm1447 was instrumental for leukemia stem cell activity.^96^ The RAS-linked abrogation of 18S-ψ609 and 18S-ψ863 disturbed aminoacyl-transfer RNA selection, altered the dynamics of the pre-translocation ribosome complex, and increased the incidence of translational miscoding and stop-codon readthrough.^97^ These results are in line with previous reports on the roles of rRNA ψ in ribosome fidelity and IRES-mediated translation initiation in yeast and mammalian cells.^98^ A dynamic, site-specific regulation of rRNA 2′-*O*-me was also measured during development in mouse,^42,48^ frog,^47^ or zebrafish.^43^ All studies reported a trend toward a general hypomethylation modification pattern during the development and overall hypermethylation in adult tissues.^42,43,47,48,99^ In a recent paper, Hafner and colleagues reported site-specific changes in rRNA 2′-*O*-me profiles during the transition of stem cells into endoderm, mesoderm, and ectoderm, and over the course of neural cortex development. Abrogation of 28S-Um3904, which was regulated during neural development, was sufficient to drive hESCs out of pluripotency and toward a neural cell fate. Polysome profiling experiment showed that 28S-Um3904 regulates the translation of mRNAs involved in the Wnt signaling that plays a crucial role in vertebrate neural development.^48^ These results clearly identify the rRNA-PTM-based ribosome heterogeneity as an important regulator of early development and differentiation.

## Ribosome biogenesis, heterogeneity, specialization, and translation regulation in MSK tissues

All MSK tissues are in a constant state of protein turnover, with a dynamic equilibrium between protein synthesis and breakdown, ensuring tissue strength and functional capacity. Besides MSK homeostasis and physiology, the regulation of protein synthesis also plays a role in MSK pathologies. Acute or chronic, MSK pathologies resulting from (over)use, traumatic events, or aging affect a large portion of the global population. The alterations in protein synthesis that occur in response to these events and injuries are primarily aimed at promoting healing, but in some instances can also fuel further tissue damage and disease development.

While data on general protein synthesis regulation in the MSK field exist, particularly for the rapidly adapting muscular system,^100^ we are missing more detailed and comprehensive investigations centered around the ribosome. In particular, research on ribosome heterogeneity and specialization in the MSK is still in its infancy. Nevertheless, studies on this topic are slowly emerging. In this review, we summarize the data related to ribosome heterogeneity in the context of skeletal muscle, bone, articular cartilage, tendon, and ligament biology and pathology.

### Bone

Protein synthesis is an essential part of bone biology. In fact, the rate of protein synthesis in adult bone in vivo is not significantly different from the protein synthesis rate in skeletal muscle.^3^ The importance of ribosomes for bone and skeletal development is underpinned by ribosomopathies, a group of genetic disorders caused by defects in ribosome biogenesis or function, that manifest with a skeletal phenotype, including short stature, craniofacial defects, limb malformations, bone marrow failure, and others.^101^

During differentiation, mesenchymal progenitor cells undergo a phase of active proliferation, coupled with the increased expression of lineage-specific factors and withdrawal from the cell cycle.^102^ This process is tightly coupled with the regulation of ribosome biogenesis.^103^ Neben and colleagues examined the regulation of the rDNA transcription during the differentiation of MC3T3-E1 mouse calvarial osteoprogenitor cells. Upon osteoinduction, osteoprogenitor cells transiently decreased rDNA transcription, resulting in a lower number of ribosomes and a reduction of total protein synthesis.^104^ Ribosome heterogeneity was not investigated in this study; however, it would be interesting to examine its role in osteoblast differentiation. In *Rpl38* (eL38) mutant mouse embryos, RPL38 was shown to regulate the formation of 80S complexes on specific Hox mRNAs, thus controlling their translation. This had important consequences for tissue-specific patterning and the homeotic transformations of the axial skeleton.^105^ Furthermore, Hafner and colleagues reported site-specific changes in rRNA 2′-*O*-me profiles during the tri-lineage differentiation of human ESCs into endoderm, mesoderm, and ectoderm. The abrogation of 2′-*O*-me at 28S-Um3904 impacted the translation of mRNAs involved in the Wnt signaling pathway, which plays a crucial role in vertebrate development.^48^ SNORA7A (predicted to guide 28S-ψ1569 and 28S-ψ1779) was found to be highly expressed in undifferentiated human umbilical cord blood-derived mesenchymal stromal cells (uMSCs) in vitro and was positively correlated with the uMSCs’ proliferative capacity and pluripotency.^106^ Ectopic expression of *SNORA7A* promoted uMSC self-renewal and regulated the expression of proliferation- and pluripotency-related genes, while inhibition had the opposite effect. Experiments with mutant forms of *SNORA7A* incapable of interacting with DKC1 and forming the snoRNP complex, abolished its effects on uMSCs self-renewal. This indicated that snoRNP formation and rRNA modifications are essential for SNORA7A-mediated functions in uMSCs. Lastly, the effect of SNORA7A on uMSC osteogenic differentiation was evaluated. *SNORA7A* overexpression significantly inhibited the formation of calcified nodules in uMSC and suppressed the expression of osteogenic marker genes, while its inhibition improved osteogenic differentiation.^106^

A study analyzing biopsies of non-weight-bearing iliac and weight-bearing femoral postmenopausal bone across BMD varying from normal to osteoporotic, found a subset of snoRNAs which correlated with BMD.^107^ In the iliac bone, SNORD44 (predicted to guide 18S-Am166) and SNORD48 (predicted to guide 28S-Cm2279)^108^ negatively correlated with BMD.^107^ In the femoral bone, another set of 12 snoRNAs correlated with BMD was identified, including SNORA3b (28S-ψ3899, 28S-ψ3938), SNORA7a and 7b (28S-ψ1569, 28S-ψ1779), SNORA54 (28S-ψ3801, 28S-ψ4539), SNORA58 (28S-ψ3823), SNORD75 (28S-Cm4032), SNORD80 (18S-Am1521, 28S-Gm1612), SNORD82 (18S-Am1678), and SNORD105b (18S-Um799). Others, such as SNORD23, SNORD114-1, and SNORD117, have no predicted rRNA targets or have functions outside of rRNA PTMs.^108^ Interestingly, levels of 28S rRNA showed a positive correlation with BMD.^107^ The link between BMD and rRNAs/ribosomes is further supported by the fact that RUNX2, a fundamental transcription factor for bone development, regulates rRNA expression during osteoblast lineage progression.^103,109^

### Skeletal muscle

Skeletal muscle occupies 35%-45% of the body mass of a healthy adult individual,^110,111^ and it is an extremely dynamic tissue capable of changing its size, composition, and metabolic characteristics in response to environmental and physiological stimuli. To adapt to resistance exercise training, muscle progressively accumulates protein mass, leading to hypertrophy. Conversely, under chronic conditions of energy deprivation, unloading, and/or disease, muscle undergoes rapid atrophy. A critical determinant of muscle mass is the ratio of protein synthesis to protein breakdown. As ribosomes stand at the center of protein synthesis, they are well recognized and studied in the muscle field.^110,112,113^ However, while most of the studies focused on the ribosome biogenesis and its regulation in muscle cell differentiation and hypertrophy,^110,113–115^ only a few had explored ribosome heterogeneity or specialization. Nevertheless, studies on this topic are emerging and provide valuable insights.

A recent study performed a proteomic analysis of ribosomal fractions purified from 14 adult mouse tissues and generated a comprehensive map of the RP-based heterogeneity of ribosomal composition in different organs and tissues in mice.^51^ The quadriceps muscle was among the analyzed tissues, thus providing data on RP-mediated ribosome heterogeneity in skeletal muscle. The data show that compared to all other tissues, the ribosomes of skeletal muscle are significantly enriched in a paralogue RPL3L (uL3L). RPS30 (eS30, Fau) was also enriched in skeletal muscle ribosomes, however, to a lesser extent than RPL3L. On the other hand, RPS26 (eS26) was less abundant, and RPS15 (uS19) was not detected at all.^51^

The tissue-specific expression of the RPL3L paralogue in skeletal muscle (and the heart) was shown previously at the level of mRNA,^116–118^ corroborating the riboproteome data.^51^ One of these studies investigated the expression of RP genes in adult mouse plantaris muscle in response to hypertrophic stimulation. Interestingly, RPL3 and RPL3L were the only two RPs whose expression changed upon stimulation—the *Rpl3l* mRNA levels decreased by 82%, while the *Rpl3* expression significantly increased.^117^ Such compensatory RPL3/RPL3L expression was also observed in CMs.^119^ The function of RPL3L as a negative regulator of muscle growth was further tested by expressing RPL3L in C2C12 myogenic cells during myotube formation. This significantly decreased myotube size as a result of decreased myoblast fusion.^117^ In line with these data, suggesting that RPL3L inhibits satellite cell fusion and hypertrophic growth, are microarray results showing that *Rpl3l* expression in muscle is very low during the early stage of postnatal development,^120^ a period characterized by a robust increase in muscle fiber size and myonuclear number as a result of significant satellite cell fusion.^121^ In the later phases of postnatal development, when the muscle growth gradually abates, the expression of *Rpl3l* gradually increases.^120,121^ The RPL3/RPL3L expression pattern is deregulated in the skeletal muscle of patients with DMD,^122–124^ a genetic myopathic disorder characterized by progressive muscle weakness and degeneration. Analysis of protein lysates of the tibialis anterior (TA) isolated from 1- to 56-d-old mice showed a significantly faster decline in RPL3 expression during the postnatal development in dystrophic *mdx* mice. The expression of RPL3L, however, did not increase until the late stage of development.^125^ Differences in RPL3/RPL3L expression patterns between the healthy and dystrophic *mdx* mice were also accompanied by alterations in muscle structure. While the healthy mice exhibited a compact, organized structure with no central nucleation and a small amount of fibrotic tissue at the 8-wk timepoint, clusters of small, centrally nucleated fibers, and large areas of fibrotic tissue were observed in *mdx* mice. *Rpl3l* knockdown significantly improved muscle function, specifically muscle strength and resistance to fatigue, in both healthy and *mdx* mice.^125^ Lastly, *Rpl3l* expression was also decreased during the first days of skeletal muscle regeneration following cardiotoxin injection.^126^

Nevertheless, the mechanisms of how exactly the RPL3L-mediated ribosome heterogeneity affects skeletal muscle biology remain to be elucidated. RPL3L shares 80% sequence homology with RPL3; however, the remaining 20% difference may allow RPL3L to fine-tune ribosome structure and function. Data from *E coli* show that RPL3 initiates the assembly of the 50S subunit^127^ and is indispensable for the formation of the peptidyl transferase center.^128^ In addition, it was suggested that RPL3 functions as an allosteric switch coordinating the binding of elongation factors to the ribosome. By gatekeeping the A site, RPL3 promoted the synchronization of aminoacyl-tRNA binding and translocation.^129^ A mutation in the *RPL3* gene affected the ribosomes’ aminoacyl-tRNA affinity, peptidyl transferase activity, and translation accuracy, specifically the frameshifting efficiency in yeast.^129^ Furthermore, data from the heart might offer insights into possible mechanisms of RPL3L-mediated ribosome specialization. In a recent study*, Rpl3l*^−/−^ mice were generated to investigate the function of RPL3L in heart biology. In a compensatory mechanism similar to the one observed in skeletal muscle upon hypertrophic stimulation,^117^  *Rpl3l* knockout led to increased expression of *Rpl3* and the formation of RPL3-containing ribosomes atypical for adult heart tissue. The ablation of RPL3L-containing ribosomes then led to ribosomal stalling at Ala/Pro codons, delaying translational elongation, and causing ribosomal collisions. While the altered translation elongation dynamics affected the entire transcriptome, the effects were most pronounced for transcripts related to cardiac muscle contraction and dilated cardiomyopathy.^130^ These data suggest that RPL3L affects ribosome translation elongation dynamics, which are important for cardiac function.^130^ It is important to mention, however, that others failed to measure the effects of RPL3L on translation elongation and efficiency.^119,131^ Differences in mouse strains and developmental stages may account for these discrepancies; nevertheless, further research is necessary to decipher the roles of RLP3L in translation. In a different study, RPL3L depletion in CMs also led to an increase in CM-atypical RPL3-containing ribosomes. These ribosomes exhibited increased interactions with mitochondria, accompanied by a significant increase in ATP levels. This suggests that the ribosome/mitochondria interaction might play a role in fine-tuning mitochondrial activity.^119^ Altogether, it is clear that RPL3/RPL3L-mediated ribosome heterogeneity plays a role in muscle biology; however, the precise functional consequences remain to be elucidated.

Anabolic signaling was also shown to regulate the phosphorylation of RPS6 (eS6) in skeletal muscle.^132–134^ This phosphorylation is catalyzed by p70 S6 kinase, a component of the mechanistic target of rapamycin complex 1 (mTORC1) pathway with well-known functions in promoting skeletal muscle hypertrophy.^135,136^ While initially it was suggested that RPS6 phosphorylation regulates the translation of mRNAs harboring a 5′-TOP sequence,^137,138^ more recent data indicate that it is not essential.^139,140^ While the downstream effects are yet to be understood, the ablation of RPS6 phosphorylation decreased skeletal muscle mass and strength through its role in the regulation of myofiber growth and energy content.^141^

Alternative splicing is another source of ribosome heterogeneity in skeletal muscle. For the RPS24 (eS24), there are at least three splicing variants that differ in their C-terminal amino acids.^67,142^ One of the splicing variants (termed ex4:22 bp/18 bp, but various nomenclature is used in the literature) is highly expressed in skeletal muscle tissue, constituting 90% of all *RPS24* transcripts.^67^ Future studies are necessary to investigate whether the muscle-enriched RPS24 isoform allows for ribosome functional specialization. Nevertheless, studies from other fields suggest this might be the case. For example, RPS24 alternative splicing and isoform switching were reported in glioma,^67^ or in hypoxic conditions, when the increased expression of the longer and more stable isoform of RPS24 promoted cell survival and growth.^70^

Moving to rRNA-mediated ribosome heterogeneity, a recent paper investigated the dynamics of rRNA 2′-*O*-me in skeletal muscle in response to growth stimuli in vivo. Mechanical overload (MOV) was applied for 3, 7, and 14 d and was followed by rRNA 2′-*O*-me profiling of plantaris muscle in mice. Compared to the sham group, MOV induced transient and site-specific changes in the rRNA 2′-*O*-me profile. Further analysis of myofiber-specific rRNAs using the HSA-RiboTag mouse model and 7-d MOV led to the identification of a subset of the rRNA 2′-*O*-me that were regulated in whole tissue as well as fibers.^143^

The loss of protein homeostasis is one of the hallmarks of aging. The ability of skeletal muscle to respond to a hypertrophic stimuli is compromised in older individuals as a result of dysfunctional ribosome biogenesis, particularly at the level of rDNA transcription.^144^ More nuanced changes in translation characteristics, specifically alterations in translation initiation, efficiency, and a decline in translation fidelity, were also reported in aging skeletal muscle in mice and rats.^145–147^ Two most studied anti-aging interventions, caloric restriction and rapamycin treatment, were shown to regulate translation efficiency in mouse geriatric muscle and partially restore aging-associated translational dysregulation. Caloric restriction restored stop-codon readthrough and translation of downstream ORFs, while long-term rapamycin treatment primarily affected the translation from uORFs and was able to partially revert aging-induced changes in ribosome interactions with 5′ UTRs.^145^ Altogether, these data indicate that age-associated changes in translation contribute to aging. Nevertheless, the precise mechanisms behind this (de)regulation are unknown. It would be relevant to investigate whether ribosome heterogeneity and specialization play a role in altered translation characteristics in aging skeletal muscles. For example, a study in *D melanogaster* showed that RpS28 (eS28) isoforms RpS28a and RpS28-like are preferentially expressed in the germline, a tissue resistant to aging, and that their expression significantly declines in skeletal muscle during aging. Muscle-specific overexpression of *RpS28a* to levels similar to those in the germline decreased early mortality and increased the protein levels of a subset of proteins, some of which were germline-enriched and/or have anti-aging functions. These findings indicate a role for specialized ribosomes during muscle aging.^148^

### Articular cartilage, ligaments, and tendons

Articular cartilage protects subchondral bone from mechanical damage and provides a smooth, lubricated surface for articulation.^149^ These mechanical properties arise from a high water content, regional and zonal composition, and the organization of ECM.^149,150^ Articular cartilage ECM is rich in proteins,^149,151^ all of which are produced by chondrocytes, the only cell type present in articular cartilage.^152^ Chondrocytes also play a critical role in endochondral ossification, whether prenatally during bone formation,^153,154^ or postnatally by enabling the longitudinal growth of long bones.^155^ The equilibrium between anabolic and catabolic processes is critical for chondrocytes’ function. Recent evidence demonstrates that ribosomes and ribosome-mediated translation regulation play important roles in maintaining chondrocytes’ homeostasis, and their deregulation leads to cartilage pathologies.^4^

The significance of ribosomes and protein synthesis regulation for chondrocytes and cartilage biology is highlighted by the fact that ribosome biogenesis and chondrocytes’ translation activity are tightly regulated during the course of chondrogenic differentiation, as demonstrated by two recent studies utilizing the mouse chondrogenic cell line mATDC5.^156,157^ In the early stages of chondrogenic differentiation, the transcription factor SOX9 (sex determining region Y-box 9), the master regulator of chondrogenic differentiation, not only induced ribosome biogenesis but also regulated the activity of the chondrocytes’ ribosomal pool.^157^ It was suggested that this is to prepare the chondroprogenitors for the demanding proliferative phase and subsequent cartilage ECM production.^157^ The expression of snoRNAs, as well as the rRNA PTM-catalyzing enzymes FBL and DKC1, was also dynamically regulated, suggesting that ribosome heterogeneity might play a role in chondrogenic differentiation.^156^ To support this, site-specific changes in rRNA ψ profiles were reported during chondrogenic differentiation in human bone marrow stem cells.^89^

Bone Morphogenetic Protein 7, a morphogen crucial for cartilage maintenance and repair by regulating chondrocyte activity, differentiation, proliferation, and ECM production,^158^ was also shown to promote ribosome biogenesis and chondrocytes’ translation capacity in the chondrocytic cell line SW1353 and in human primary chondrocytes.^159^ TGF-β, a pleiotropic cytokine with a role in cartilage homeostasis but also pathology, controlling cell proliferation, tissue repair, and inflammation,^160^ was also investigated. In chondrocytic SW1353 cells and human primary chondrocytes, TGF-β2 induced total protein synthesis and, more specifically, the cap-mediated translation.^161^ The increase in cap-mediated translation was accompanied by the inhibition of IRES-mediated translation initiation. The analysis of the ribosomal proteome in chondrocytic cells treated with TGF-β2 showed changes in the stoichiometry of several RPs as well as RAPs, many of which have functions in translation initiation.^162–168^ For example, the RNA helicase eIF4A3 and several members of the heterogeneous ribonuclear protein (HNRNP) family showed decreased association with the ribosomes of TGF-β2-treated cells. This could be important, as eIF4A aids the 40S ribosomal subunit to pass the secondary structures within the mRNAs 5′ UTR,^169^ and RNA-binding HNRNPs are known IRES-transacting factors.^162–165^ Overall, these results follow the observed increase in total protein translation and a relative decrease in IRES-mediated translation in TGF-β2-treated chondrocytes.^161^ Finally, site-specific changes in rRNA 2′-*O*-me and ψ profiles were measured in response to TGF-β2 treatment. One of the differentially modified sites was 18S-Gm1447. The differential modification of 18S-Gm1447 was also reported in acute myeloid leukemia, where it promoted leukemia stem cell activity and phenotype.^96^ Furthermore, it is located in very close proximity to RPS20 of the 40S subunit. RPS20 binds canonical translation initiation factors and can also interact with IRES elements.^170–172^ Interestingly, RPS20 was one of the RPs that exhibited altered stoichiometry in TGF-β2-treated chondrocytes. Altogether, this might suggest that the 18S-Gm1447 modification status could affect the stoichiometry of RPS20, which could lead to ribosome functional specialization.

Ribosomes were also investigated in the context of OA. Osteoarthritis is a degenerative joint disorder characterized by the degeneration of the articular cartilage, which is a result of the “activation” of quiescent chondrocytes.^173^ This activation, to some extent, resembles the chondrogenic phase of the endochondral ossification process in growth plates during bone development.^174^ However, in the context of fully developed, adult articular cartilage, it has detrimental consequences. In OA, “activated” chondrocytes undergo a phenotypic shift^175^ fueled by substantial shifts in their proteome and secretome.^176^ In the course of OA development and progression, chondrocytes exhibit (de)regulated expression of RPs,^177^ rRNAs,^158,159,178^ as well as snoRNAs,^178–183^ thus modifying their translation characteristics and proteome.^159,178,184–189^

Ribosome heterogeneity was also implicated in OA.^54,55^ Using an established OA in vitro model that emphasizes the fibrochondrocyte OA phenotype,^190^ it was demonstrated that the OA microenvironment instigates site-specific changes in rRNA 2′-*O*-me^55^ and ψ profiles.^54^ Two OA-associated rRNA PTMs, 5.8S-Um14^55^ and 28S-ψ4966,^54^ were selected for functional validation. The 5.8S-U14 nucleotide is located within the 28S-5.8S rRNA junction, and its 2′-*O*-me status affects the conformational state of 5.8S and its interaction with 28S.^191^ 5.8S-Um14 belongs to variable modifications,^41^ suggesting that it might fine-tune ribosome function. Manipulation of the OA-associated 5.8S-Um14 by depleting its guide snoRNA, *SNORD71* in chondrocytic cells led to a significant increase in collagen type I alpha 1 protein levels, while the total abundance of *COL1A1* mRNA was unchanged. Analysis of the polysomal profile suggested the increased efficiency of *COL1A1* translation by ribosomes in SNORD71-depleted cells.^55^ While further analysis is necessary to clarify the mechanisms underlying 5.8S-Um14-mediated translation regulation of *COL1A1* mRNA, some indications can be found in the literature. The 5′ UTR of *COL1A1* mRNA contains a stem-loop structure and short uORFs.^192,193^ The evolutionary conserved *COL1A1* 5′ stem-loop is located about 75 nucleotides downstream of the 5′ cap, and it encompasses the translation initiation codon. The stability of the *COL1A1* 5′ stem-loop is not sufficient to completely block ribosomes during scanning,^194^ nevertheless, experiments with reporters containing the *COL1A1* 5′ stem-loop demonstrated its ability to repress translation in mouse Mov-13 fibroblasts in vitro*.*^192^ The second OA-associated rRNA PTM that was functionally validated by knocking out its guide, *SNORA33*, was 28S-ψ4966.^54^ Ribosomes lacking 28S-ψ4966 exhibited changes in the stoichiometry of RPS26 as well as many RAPs, including several well-known translation factors and proteins involved in ribosome stalling and recycling pathways.^54^ In terms of the effects on the cellular proteome, the ablation of 28S-ψ4966 decreased the expression of several inflammation-attenuating factors,^54^ including programmed cell death protein 11 (PDCD11, also called NFBP)^195–197^ and α-2-macroglobulin (A2M).^198^ Importantly, changes in the cellular proteome detected in *SNORD71-* and *SNORA33*-depleted cells were distinct and specific to the depleted snoRNA, strongly suggesting that each modification has distinct functions.^54,55^ This is particularly important as the interpretation of data presented by some studies on ribosome heterogeneity and specialization has been debated recently.^199^ The studies in question depleted different ribosomal components; however, they reported highly overlapping translational effects.^199^ This suggested that the observed proteomic differences could be the result of reduced ribosome numbers (ribosome concentration theory^11^), rather than ribosome specialization. No differences in proliferation, the rate of total protein synthesis, polysomal profiles, nor overlapping effects on cellular proteomes were reported in the 5.8S-Um14 and 28S-ψ4966 studies, supporting the conclusions about ribosome functional specialization in OA.

This is further supported by previously reported translation regulation at the level of individual mRNAs in OA. Ribosome profiling experiments showed that IL-1β stimulation induced rapid alterations in the translation rates of specific transcripts associated with OA and inflammation, such as *NFκB1, TNF Alpha Induced Protein 2 (TNFAIP2), MMP13*, and chemokines *CCL2*, or *CCL7.*^187^ The translation regulation of specific genes in response to inflammatory stimulation was also demonstrated in rat articular chondrocytes treated with IL-1β.^200^ Fibronectin 1 (Fn1) was the most striking example. In the non-treated rat articular chondrocytes, *Fn1* mRNA levels were high, however, its translation was strongly suppressed (only 1% of *Fn1* mRNA was translated), resulting in low FN1 protein levels. However, when exposed to IL-1β, the translation suppression of *Fn1* mRNA was relieved, leading to a strong increase in FN1 protein abundance.^200^ Many other mRNAs (almost 16% of all detected genes), including *Col2a1* and *Acan*, also showed low polysomal representation compared to their total mRNA levels, which is indicative of translational repression. Further inspection of *Fn1* and two other translationally regulated transcripts, *Sulf1* and *Nr4a1*, revealed that their 5′ UTRs are important determinants of the IL-1β-mediated translation induction. In fact, putative IRES elements were identified within the *FN1* and *Nr4a1* genes in a recent IRES element systematic screening in the human genome.^201^ IRES elements were also identified in several OA-relevant genes including *mTOR, IGF1R, LEF1, cJUN, FGF1, FGF2, HSPA1A, RUNX1, RUNX2, VEGFA, CTSL, P53*, or *XIAP*,^201^ suggesting that IRES-mediated translation might play a role in OA development and progression. In support of this, the exposure of primary chondrocytes to an OA microenvironment increased the rate of IRES-mediated translation.^55^

Data on rRNA PTM-mediated ribosome heterogeneity and specialization in OA are further supported by studies reporting the differential expression of snoRNAs in lesional and non-lesional cartilage from patients with end-stage OA, in in vitro cultured primary human chondrocytes exposed to OA synovial fluid or IL-1β,^179^ mouse and equine OA models.^180,183^ Aging, a major risk factor for OA but also many other MSK diseases, also seems to (de)regulate snoRNA expression as shown in human^179^ and equine cartilage,^202^ mouse joints,^180^ equine cartilage or in an in vitro replicative aging model.^203,204^ SnoRNAs might even serve as biomarkers of cartilage damage and OA, as suggested by data reported in humans,^205^ mice,^180^ or horses.^182^

Excessive mechanical loading in in vitro cultured primary mouse chondrocytes, as well as human and mouse cartilage in vivo, *led to* a decreased cellular abundance of RPL35 (uL29).^206^ RPL35 knockdown in mechanically loaded chondrocytes in vitro induced senescence and decreased the expression of anabolic markers. Finally, intra-articular overexpression of *RPL35* in mice with OA induced by destabilization of the medial meniscus and compression loading significantly decreased OA severity.^206^ Even though further studies are needed to establish whether this effect of RPL35 on cellular senescence and the chondrocyte phenotype is dependent on its differential ribosomal stoichiometry, or extra-ribosomal functions, it is another indication of ribosome heterogeneity and specialization in OA.

Lastly, ribosomes and translation are yet to be investigated in ligaments and tendons. Nevertheless, (de)regulated expression of snoRNAs was reported in anterior cruciate ligament samples from patients with end-stage OA,^207^ and in aging human Achilles tendons,^208^ suggesting that ribosomes might play a role in the (patho)biology of these tissues.

## New technologies for advancing ribosome research

High-quality ribosome isolation is foundational to ribosome research. While the traditional methods, such as the sucrose cushion^209^ or gradient ultracentrifugation^210^ and immunopurification (eg, Ribo-Flag immunoprecipitation^79^ or Ribo-Tag^211^) represent gold standards; they are, however, labor-intensive, lengthy, and expensive. Furthermore, they require substantial input material and specialized equipment, posing a limitation for ribosome-centered research. Recently developed methods—including RAPPL,^212^ PAPIDASH,^27^ ARC-MS,^213^ or Ribo Mega-SEC^214^—represent an alternative to the traditional methods and hold great potential. RAPPL (RNA affinity purification using poly-lysine) enables rapid, cost-effective purification applicable across species and sample types and is compatible with follow-up structural and functional studies.^212^ RAPIDASH enriches ribosomes and RAPs without the need for genetic manipulation,^27^ while active ribosome capture-MS relies on “click” chemistry to identify RAPs recruited to translationally active ribosomes at the early stages of translation.^213^ An alternative to sucrose gradient ultracentrifugation, especially when comparing several cell types, tissues, or conditions, is Ribo Mega-SEC, which utilizes size-exclusion chromatography and ultra-high performance liquid chromatography to isolate polysomes and ribosomal subunits, and it is compatible with downstream MS, electron microscopy, and structural analyses.^214^

Following ribosome isolation, MS represents a cornerstone for investigating RP- and RAP-based ribosome heterogeneity. Bottom-up proteomics, where proteins are enzymatically digested into peptides, is widely used due to its robustness, sensitivity, and scalability. However, a key challenge associated with bottom-up proteomics is that it lacks the ability to resolve proteoforms. The top-down proteomics, where intact proteins are directly analyzed by MS without prior digestion, offers an alternative approach that enables the direct identification of proteoforms, isoforms, and protein modifications. Furthermore, unlike bottom-up proteomics, it is compatible with native MS and conformational analysis, which are crucial for linking ribosome compositional heterogeneity with functional specialization and translational regulation.^215–217^ Finally, emerging microfluidic MS platforms and single-cell proteomics are further expanding the boundaries of ribosome analysis in rare, low-input samples or individual cell populations.^218,219^

In terms of rRNA-mediated ribosome heterogeneity, the development of RiboMethSeq and HydraPsiSeq—high-throughput sequencing-based methods to map and quantify rRNA 2′-*O*-me and ψ—represented a milestone and greatly contributed to key discoveries in the field.^89,92^ On the other hand, the analysis of rRNA sequence variation remains a challenge due to several technical difficulties. Nevertheless, long-read sequencing technologies, including nanopore or single-molecule real-time sequencing, combined with advanced computational approaches, should help resolve these complexities.^83,86^

Cryogenic electron microscopy has revolutionized ribosome research by enabling high-resolution, near-native 3D visualization of ribosomal structures and dynamics without the need for crystallization. Cryo-EM allows for the identification of protein and rRNA modifications and reveals interactions between ribosomal components as well as surrounding solvent molecules. When integrated with downstream MS, RNA-seq, or PTM mapping, it enables correlating structural and functional ribosomal characteristics all within one sample.^220,221^ Moreover, cryo-EM and single-molecule resolution techniques coupled with advanced data analysis approaches are poised to uncover combinatorial and co-occurring patterns and networks of various levels of ribosome heterogeneity and structural variants, their complex regulation, and their consequences for ribosome function.^218–220,222,223^ We have a very limited understanding of the interconnected networks of individual layers of ribosome heterogeneity, the mechanisms by which ribosomes sense, communicate, integrate, synchronize, and respond to differences in their composition at a global level, and how this affects ribosome dynamics and function. A work from Timsit and colleagues has offered fascinating insights into ribosome dynamics, movement, intermolecular interactions, and signaling during translation. They demonstrated the existence of RP networks mediating ribosome signaling and described the “electrostatic domino” effect by which RP networks synchronize and coordinate ribosomal dynamics during translocation.^223^ These types of data provide a conceptual framework for understanding ribosome heterogeneity and functional specialization.

To uncover the effects of ribosome heterogeneity on the cellular proteome, cross-genome analyses of translation dynamics are essential. One of the key methodologies that has transformed our understanding of translation regulation is ribosome profiling (also called Ribo-Seq). Since its introduction back in 2009,^224^ the method has evolved considerably. It is now possible to study ribosome mRNA scanning and translation initiation, elongation at various conformations, and to assess compartmentalized translation and the dynamics of cotranslational factors bound to the ribosomes,^225–227^ or perform ribosome profiling at a single-cell resolution (scRibo-seq).^228^ Finally, an alternative to ribosome and polysome profiling that uses standard RNA-seq and quantification to measure transcriptome-wide ribosome association in single cells, Ribo–STAMP (surveying targets by APOBEC-mediated profiling) has been described. It can distinguish genes with varying levels of ribosome occupancy and detect translational perturbations and dynamic translational responses.^229^

**Figure 3:**
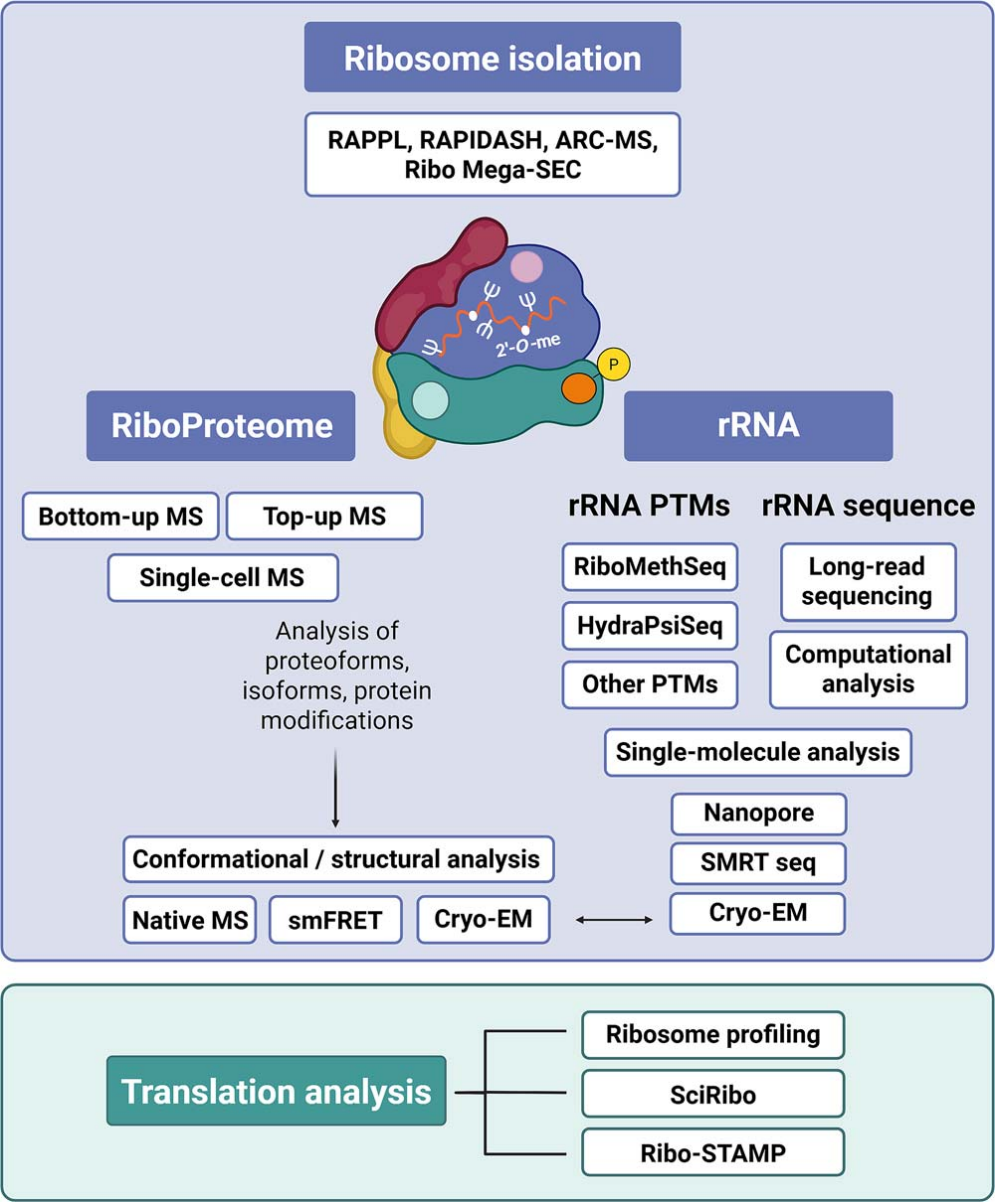
Emerging technologies and methodologies in ribosome-centered research. Abbreviations: ARC-MS, active ribosome capture-MS; cryo-EM, cryo-electron microscopy; MS, mass spectrometry; PTM, post-transcriptional modification; RAPIDASH, RAP identification by affinity to sulfhydryl-charged resin; RAPPL, RNA affinity purification using poly-lysine; ribo–STAMP, ribosome surveying targets by APOBEC-mediated profiling; rRNA, ribosomal RNA; SEC, size-exclusion chromatography; sciRibo, single-cell resolution ribosome profiling; smFRET, single-molecule Förster resonance energy transfer; SMRT seq, single-molecule real-time sequencing. Figure created using BioRender.

Together, these emerging technologies and computational approaches provide unprecedented opportunities to uncover the full scope of ribosome heterogeneity and its consequences for ribosome functional specialization in MSK (patho)biology (Figure 3).

## Conclusions

**Figure 4:**
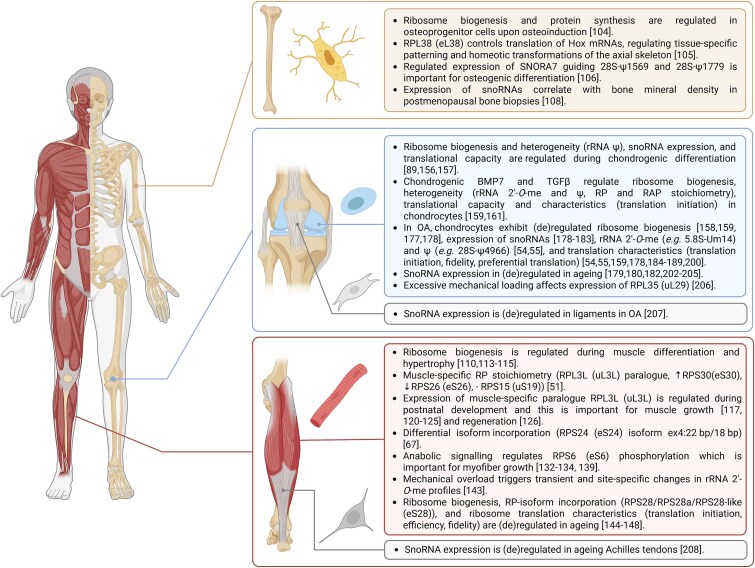
Generalized summary of the key findings on ribosome heterogeneity and specialization in MSK (patho)biology. Abbreviations: 2′-*O*-me, ribose 2′-*O*-methylation; OA, osteoarthritis; RAP, ribosome-associated protein; RP, ribosomal protein; rRNA, ribosomal RNA; snoRNA, small nucleolar RNA; ψ, pseudouridylation. Figure created using BioRender.

Over the past two decades, research into ribosome heterogeneity and specialization has gained considerable momentum across various fields, with a growing body of evidence highlighting its significance. Although the MSK field has yet to fully embrace this emerging research area, accumulating data suggest that ribosome heterogeneity and specialization play important roles in MSK biology and pathology—supporting development, maintaining homeostasis, and facilitating cellular and tissue functions, but also driving pathological changes and disease progression (Figure 4).

Nevertheless, this is only the beginning. To fully grasp the scope and impact of ribosome heterogeneity and specialization in MSK (patho)biology, further in-depth ribosome-focused studies are necessary. A comprehensive understanding of the dynamics and regulation of ribosome heterogeneity—during development, differentiation, growth, in response to various stimuli, aging, or in diseases—is still lacking. Moreover, functional validation and mechanistic insights into how these heterogeneous ribosomes exert their specialized functions are needed. While there is still a lot to uncover, key technologies and methodologies are rapidly advancing, offering promising tools to address these fundamental questions.
